# Editorial: Microbial Taxonomy, Phylogeny and Biodiversity

**DOI:** 10.3389/fmicb.2019.01324

**Published:** 2019-06-19

**Authors:** Jesús L. Romalde, Sabela Balboa, Antonio Ventosa

**Affiliations:** ^1^Department of Microbiology and Parasitology, CIBUS-Faculty of Biology, Universidade de Santiago de Compostela, Santiago de Compostela, Spain; ^2^Department of Microbiology and Parasitology, Faculty of Pharmacy, University of Sevilla, Sevilla, Spain

**Keywords:** microbial systematics, taxonomy, phylogeny, diversity, genomics

The great diversity of microbial life is the remaining major reservoir of unknown biological diversity on Earth. To understand this vast, but largely unperceived diversity with its untapped genetic, enzymatic and industrial potential, microbial systematics is undergoing a revolutionary change in its approach to describe novel taxa based on genomic/envirogenomic information (Rosselló-Móra and Whitman, 2019).

The characterization of an organism is no longer bounded by methodological barriers, and it is now possible to fully sequence the whole genome of a strain to study individual genes, or to examine the genetic information by using different techniques (Mahato et al., 2017). In fact, the application of genomics is helping not only to provide a better understanding of the boundaries of genera and higher levels of classification, but also to refine our definition of the species concept. In addition, increased understanding of phylogeny is allowing to predict the genetic potential of microorganisms for biotechnological applications and adaptation to environmental changes.

The present Research Topic on “Microbial Taxonomy, Phylogeny and Biodiversity” compiles 23 articles covering the use of genomic sequence data in microbial taxonomy and systematics, including evolutionary relatedness of microorganisms; application of comparative genomics in systematic studies; or metagenomic, metatranscriptomic or metaproteomic approaches for biodiversity studies, among others.

Two of the articles introduce novel methodological proposals as a genome-inferred biology or a software to infer pangenomic phylogenies using selected orthologous genes. Thus, Palmer et al. propose to utilize a holistic biological perspective by identifying genome-based characteristics in metabolic networks for taxonomic levels higher than species, in order to link taxonomy and evolution to ecology and appearance of relevant differences during speciation. On the other hand, Vinuesa et al. develope the software package GET_PHYLOMARKERS, a pipeline to identify and select optimal orthologous genes as markers to infer pangenome phylogenies, with the aim of avoid loci with undesirable properties for phylogenetic reconstruction. The genus *Stenotrophomonas* was employed by these authors to validate the protocol identifying also several misclassified RefSeq genome sequences.

A group of 5 manuscripts constitute a section dedicated to the study of the microbial diversity in different environments using metagenomic approaches. Oueriaghli et al. analyze the changes in the community composition associated to physico-chemical variations in a hypersaline environment, demonstrating that salinity and oxygen are key paramters for the dominance of some phyla in the community. Rubio-Portillo et al. study the differences in the microbiome and pathobiome associated to *Cladocora caespitosa*, a coral species abundant in the Mediterranean Sea and heavily affected by the global warming, and Berlanga et al. analyze the functional stability and dynamics of bacterial populations in microbial mats from the Camargue wetlands of Rhone delta (France). Two of the articles investigate the gut microbiome of pigs and mice to determine its role in the feed efficiency or the effects of chronic infectious, respectively (Tan et al.; Bao et al.).

Seven articles are devoted to the study of taxonomic relationships at genus level in different groups of microorganisms, including Gram-positive and Gram-negative bacteria or archaea. The need of a taxonomic rearrangement of the family *Geodermatophilaceae* is indicated by Montero-Calasanz et al. who describe a novel genus, *Klenkia*, together with the reassignation and emendation of numerous species of this group. Gupta et al. propose the division of the genus *Mycobacterium* in five different genera on the basis of phylogenomic and comparative genomic studies and, similarly, Pérez-Cataluña et al. suggest the division of the genus *Arcobacter* in seven different taxa. On the other hand, Lorén et al. use the generalized mixed Yule coalescent (GMYC) method to determine the species delineation, the phylogenetic relationships as well as to stablish the temporal divergence model in the genus *Aeromonas*, while Beukes et al. evaluate the generic boundaries of the genus *Burkholderia* and related taxa clarifying their evolutionary relationships. The description of new genospecies within the genus *Pseudomonas* through the analysis of more than 370 genomes is performed by Tran et al. who also indicate the misidentification of a large number of genomes mainly from *P. syringae* or *P. fluorescens*. Within archaea, de la Haba et al. perform genomic and polar lipid profiles analyses for the genus *Halorubrum*, being able to establish cutoff values for species delineation using a MLSA approach.

Pujalte et al. employ a polyphasic approach not only to describe two new species within the genus *Thalassobius* but also to carry out a taxogenomic study of the genera *Thalassobius, Shimia* and *Thalassococcus*, pointing out the difficulties for classification at the genus level within the *Roseobacter* group. The taxonomic status within the *Pseudomonas syringae* species group is analyzed by Gomila et al. demonstrating not only the misclasification of a high proportion of the strains studied, but also the possibility of 7 new species within this bacterial group. In a study of two species of *Micromonospora*, Riesco et al. define a working framework for defining species within this genus both at genomic and phenotypic levels. On the other hand, Saati-Santamaría et al. describe the new bacterial species *Pseudomonas bohemica* sp. nov. from bark beetles, indicating also its biotechnological potential as producer of bioactive substances with antimicrobial activity, whereas Sánchez-Hidalgo et al. determine the biosynthetic potential of *Amycolatopsis* strains by means of comparative genomics.

A group of articles are focused in the study of the intra-species taxonomy of different bacteria, including *Vibrio vulnificus* (Roig et al.), *Aeromonas sobria* (Gauthier et al.) or *Arcobacter cryaerophilus* (Pérez-Cataluña et al.). In these articles, updated intra-species classifications are proposed including well defined phylogenetic lineages, subclades, or genomovars most of them with epidemiological significance. Finally, the aim of the only article not using a genomic approach is to develop culture strategies for isolation of fastidious *Leptosira* serovar Hardjo and the use of molecular methods for differentiation of its two genotypes, which can be helpful for seroepidemiology and immunoprofilaxis of leptospirosis (Chideroli et al.).

In summary, the collection of papers published in this Research Topic provides an updated picture on the utility of the genomic approach on microbial systematics and biodiversity studies, presents new tools for the *in silico* analysis of phylogenetic relationships, and contributes scientific basis for future standardization of taxa descriptions. If in addition serves as incentive and encouragement to researchers and future discussions on microbial taxonomy and phylogenetics, it would fulfill more than enough its original aims.

## Author Contributions

JR led writing of the manuscript. All authors contributed to manuscript revision, read, and approved the submitted version.

### Conflict of Interest Statement

The authors declare that the research was conducted in the absence of any commercial or financial relationships that could be construed as a potential conflict of interest.
